# PagKNAT5a promotes plant growth by enhancing xylem cell elongation and secondary wall formation in poplar

**DOI:** 10.1093/hr/uhaf125

**Published:** 2025-05-07

**Authors:** Li-Chao Huang, Jian-Xin Lai, Xin Tian, Yu-Yu Li, Yu-Han Chen, Yi An, Cheng Jiang, Ning-Ning Chen, Meng-Zhu Lu, Jin Zhang

**Affiliations:** National Key Laboratory for Development and Utilization of Forest Food Resources, Plant Cell Wall Research Centre, College of Forestry and Biotechnology, Zhejiang A&F University, No.666 Wusu Street, Lin'an District, Hangzhou, Zhejiang 311300, China; Zhejiang Provincial Key Laboratory of Forest Aromatic Plants-Based Healthcare Functions, Zhejiang A&F University, No.666 Wusu Street, Lin'an District, Hangzhou, Zhejiang 311300, China; National Key Laboratory for Development and Utilization of Forest Food Resources, Plant Cell Wall Research Centre, College of Forestry and Biotechnology, Zhejiang A&F University, No.666 Wusu Street, Lin'an District, Hangzhou, Zhejiang 311300, China; National Key Laboratory for Development and Utilization of Forest Food Resources, Plant Cell Wall Research Centre, College of Forestry and Biotechnology, Zhejiang A&F University, No.666 Wusu Street, Lin'an District, Hangzhou, Zhejiang 311300, China; National Key Laboratory for Development and Utilization of Forest Food Resources, Plant Cell Wall Research Centre, College of Forestry and Biotechnology, Zhejiang A&F University, No.666 Wusu Street, Lin'an District, Hangzhou, Zhejiang 311300, China; National Key Laboratory for Development and Utilization of Forest Food Resources, Plant Cell Wall Research Centre, College of Forestry and Biotechnology, Zhejiang A&F University, No.666 Wusu Street, Lin'an District, Hangzhou, Zhejiang 311300, China; National Key Laboratory for Development and Utilization of Forest Food Resources, Plant Cell Wall Research Centre, College of Forestry and Biotechnology, Zhejiang A&F University, No.666 Wusu Street, Lin'an District, Hangzhou, Zhejiang 311300, China; National Key Laboratory for Development and Utilization of Forest Food Resources, Plant Cell Wall Research Centre, College of Forestry and Biotechnology, Zhejiang A&F University, No.666 Wusu Street, Lin'an District, Hangzhou, Zhejiang 311300, China; National Key Laboratory for Development and Utilization of Forest Food Resources, Plant Cell Wall Research Centre, College of Forestry and Biotechnology, Zhejiang A&F University, No.666 Wusu Street, Lin'an District, Hangzhou, Zhejiang 311300, China; National Key Laboratory for Development and Utilization of Forest Food Resources, Plant Cell Wall Research Centre, College of Forestry and Biotechnology, Zhejiang A&F University, No.666 Wusu Street, Lin'an District, Hangzhou, Zhejiang 311300, China

## Abstract

Investigating the regulatory mechanisms that govern plant growth is crucial for developing high-yield wood varieties. In this context, the KNOX gene family has been identified as a significant regulator of plant growth. Our study focuses on *PagKNAT5a*, a class II member of the KNOX gene family, which has been found to promote the growth of poplar. Transgenic plants overexpressing *PagKNAT5a* exhibited significant increases in both plant height and stem diameter compared to wild-type controls. Histochemical analyses revealed that these overexpression lines had elongated xylem vessels and fiber cells, which correlated with elevated auxin levels. Additionally, we observed thickened secondary cell walls (SCWs) and increased lignin content in the fiber cells of these transgenic lines. Further protein interaction assays indicated that PagKNAT5a physically interacts with MYB46, a crucial regulator of SCW biosynthesis. This interaction activates downstream secondary wall MYB-responsive elements (SMREs), leading to the upregulation of lignin biosynthesis genes driven by these *cis*-acting elements. Moreover, the increased photosynthetic rate observed in the overexpression lines is likely to significantly support overall plant development. Our findings suggest that PagKNAT5a facilitates the longitudinal elongation of vascular cells by modulating auxin levels while simultaneously promoting the radial growth of xylem tissue through the activation of the MYB46-mediated lignin biosynthesis pathway. The functional analysis of PagKNAT5a highlights its potential for improving wood yield in forestry applications.

## Introduction

Wood serves as a vital renewable resource, and understanding the molecular regulatory mechanisms governing secondary xylem development in plants is essential for improving both wood yield and quality. A multitude of transcription factors have been implicated in xylem synthesis, with members of the KNOTTED LIKE HOMEOBOX (KNOX) gene family playing significant roles [1, 2].

The KNOX gene family is a subset of the THREE AMINO-ACID LOOP EXTENSION (TALE) transcription factor family, and based on their sequence similarity, KNOX genes can be categorized into two distinct subfamilies [3]. In *Arabidopsis thaliana*, eight KNOX family members have been identified. *SHOOT KNOTTED-LIKE FROM ARABIDOPSIS THALIANA 1/BREVIPEDICELLUS* (*KNAT1/BP*), *MERISTEMLESS* (*STM*), *KNAT2*, and *KNAT6* belong to the class I KNOX genes, while *KNAT3*, *KNAT4*, *KNAT5*, and *KNAT7* belong to the class II KNOX genes [4].

In *Arabidopsis*, the class I KNOX subfamily genes are predominantly expressed in the shoot apical meristem (SAM) and are essential for the establishment and maintenance of the meristem. However, investigations have revealed that the class I KNOX genes exerted diverse effects on vascular meristem development. BP/KNAT1 facilitates xylem differentiation from the cambium [5, 6]. Whereas in poplar, *ARBORKNOX1* (*ARK1*), a homolog of *Arabidopsis STM* and *KNAT1*, is specifically expressed in the vascular cambium region and inhibits the differentiation of cambial cells into xylem cells [7]. Furthermore, *PagKNAT2/6b*, a homologous gene to *KNAT2* and *KNAT6*, is specifically expressed in developing xylem tissues and significantly represses both xylem cell differentiation and secondary cell wall (SCW) deposition processes [8]. These findings indicate that class I KNOX members negatively regulate vascular xylem formation in poplar.

The impact of class II KNOX genes on xylem development is intricately complex. Class II KNOX genes are widely expressed in plants and play a crucial role in regulating SCW synthesis [1, 9]. Limited studies have been conducted on class II KNOX genes such as *KNAT3*, *KNAT4*, and *KNAT5*; their single mutants exhibited normal vascular morphology in *Arabidopsis*. Nevertheless, overexpression of *KNAT3* resulted in the development of thickened interfascicular fiber, indicating that *KNAT3* positively regulated the SCW deposition of fiber cells [10]. Considerable efforts have been devoted to elucidating the role of the *KNAT7* gene in vascular development. The *Arabidopsis knat7* mutant exhibited an increased SCW thickness in fiber cells accompanied by elevated transcriptional levels of genes associated with lignin, cellulose, and xylan synthesis [9]. Moreover, the dominant repression of *KNAT7* led to a decrease in SCW thickness of fiber cells, suggesting that KNAT7 negatively regulates SCW synthesis [11]. This negative regulatory effect is relatively conserved in rice and cotton as well [12, 13]. In poplar, PtrKNAT7 was found to directly suppress the expression of *COUMARATE 3-HYDROXYLASE 1* (*CCoAOMT1*), a key enzyme in the lignin biosynthesis pathway. Overexpression of *PtrKNAT7* in the *Arabidopsis knat7* mutant resulted in thinner SCW [9, 14], indicating a potential negative regulatory role of *PtrKNAT7* in vascular SCW thickening. However, *NbKNAT7* overexpression plants showed thickened fiber SCWs while plants silenced for *NbKNAT7* had thinner cell walls in tobacco species [15]. Overexpression of *PtKNAT7* and *AtKNAT7* in poplar resulted in increased expressions of *CesA8*, *PAL*, *IRX9*, and *CCR* genes. Conversely, antisense suppression of *PtKNAT7* led to reduced expression levels of these genes along with a decrease in lignin content [16]. Therefore, KNAT7 appears to be a positive regulator of SCW synthesis within tobacco and poplar species.

It has been observed that class II KNOX family members exhibit extensive interactions with various transcription factors, facilitated by their broad expression across diverse plant tissue types [17–19]. The interactions of widely expressed homologs of KNAT7 with different transcription factors, leading to varied effects on SCW deposition, are crucial for the formation of secondary xylem [11–13, 15, 16]. The KNAT7-BLH6 heterodimers inhibited the expression of the HD-Zip III transcription factor *REVOLUTA*/*INTERFASCICULAR FIBERLESS 1* (*REV*/*IFL1*), which positively regulates SCW deposition [18, 20]. The interaction between MYB75 and KNAT7 results in the repression of downstream genes associated with lignin biosynthesis [21, 22]. The interaction between KNAT7 and OVATE FAMILY PROTEIN 4 (OFP4) enhances the transcriptional repression activity of KNAT7 [17]. KNAT7 interacts with NAC31 to inhibit the MYB61-CESAs pathway downstream of NAC31 [13], while KNAT3 and KNAT7 form heterodimers, which alleviated the inhibitory effect of KNAT7 [10, 19]. Therefore, the regulation of class II KNOX family members on SCW synthesis is partially mediated by their interactions with specific transcription factor in particular environmental conditions. The varying effects of the KNAT7 homologous gene on SCW synthesis in *Arabidopsis*, cotton, tobacco, and poplar [15, 16] may be attributed to its interaction with distinct cooperators within species-specific tissue contexts.

The complex and sometimes contradictory roles of *KNAT7* homologous genes across different plant species highlight the diverse functions of this gene family in regulating SCW development. The influence of specific class II KNOX members on woody xylem development should not be exclusively inferred from the functional studies of their homologous in other species. Thus, whether other class II KNOX genes are involved in the SCW synthesis of xylem elements in poplar remains to be studied. In this study, we identified *PagKNAT5a* from hybrid poplar 84K (*Populus alba* × *P. glandulosa* clone ‘84K’), which shares homology with *Arabidopsis* orthologous genes *KNAT3*, *KNAT4*, and *KNAT5*. We found that PagKNAT5a positively regulates the longitudinal elongation of xylem elements and SCW thickness of fiber cells in xylem. We investigated the role of PagKNAT5a in xylem development by focusing on its interaction with MYB46 and its effects on downstream lignin synthesis-related genes. This study provides novel insights into the potential role of KNOX genes in increasing wood production.

## Results

### *KNAT5a* is widely expressed in vascular tissues during xylem development in poplar

**Figure 1:**
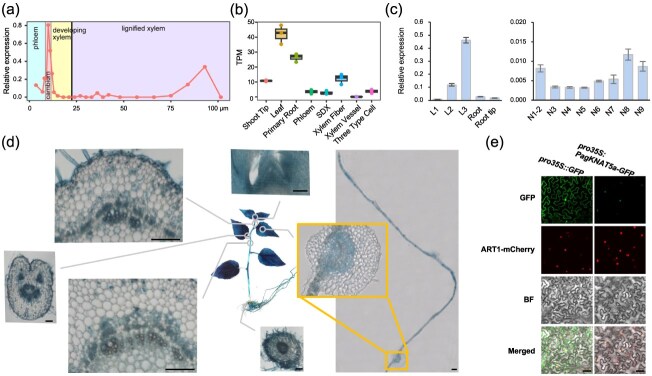
Characterization of the *PagKNAT5a* gene in poplar. (a) Expression patterns of *KNAT5a* in *P. tremula* [23]*.* (b) Expression of *KNAT5a* in different tissues including shoot tip, leaf, primary root, phloem, stem differentiating xylem (SDX), xylem fiber, xylem vessel, and three cell types (fiber, vessel, and ray cells) [24]. (c) qRT-PCR analysis of *PagKNAT5a* in various tissues from 84K poplar. Data are represented as mean ± SD (*n* = 4). L1–L3, the first to third unfolding leaves. N1–N9, the first to ninth internodes. (d) GUS staining of 1-month-old tissue culture seedlings of *proPagKNAT5a:GUS* transformants and sections of the apical bud, third stem, leaf, petiole, fifth stem, and root. Magnified image showed the midvein of the third unfolding leaf. Scale bar: 100 μm. (e) Transient expression of PagKNAT5a-eGFP fusion protein in *Nicotiana* mesophyll cells stably expressing a nuclear localization label (ART1-mCherry) [25]. Scale bar: 50 μm.

A Neighbor-Joining tree analysis was employed to explore the relationship among KNOX transcription family members in *Arabidopsis* and *Populus trichocarpa*, identifying four closely related *Populus* members in class II: *PtrKNAT5a*, *PtrKNAT5b*, *PtrKNAT3a*, and *PtrKNAT3b* (Fig. S1a). Expression data from vascular tissues of 45-year-old wild-growing aspen trees (*Populus tremula*) [23] indicated that *PtKNAT5a* is primarily expressed in the cambium and mature xylem during wood formation (Fig. 1a). Similarly, expression profiling in various tissues and cell types of 5-month-old *P. trichocarpa* Nisqually-1 plants demonstrated widespread *PtrKNAT5a* expression, with elevated transcriptional level in leaves, roots, and xylem fibers (Fig. 1b) [24]. To analyze the expression pattern of *PagKNAT5a* in 1-month-old soil-grown 84K plants, quantitative real-time PCR (qRT-PCR) detection was performed, which revealed that *PagKNAT5a* was widely expressed in poplar leaf, root, and stem (Fig. 1c). Further analysis using stable *proPagKNAT5a:GUS* transgenic lines confirmed extensive *PagKNAT5a* expression in 84K poplar, with strong β-glucuronidase (GUS) staining signals in leaves, stems, and roots. Histological sections showing predominantly GUS activity in vascular tissue including phloem, cambium, and xylem, though cambial signals were mainly localized to specific cambial cells (Fig. 1d). *In situ* PCR assays corroborated these findings, showing *PagKNAT5a* expression in phloem, cambium, and xylem, with notable concentration in cambium ray parenchyma cells (Fig. S2). Subcellular localization studies of PagKNAT5a-eGFP in *Nicotiana* mesophyll cells indicated nuclear localization (Fig. 1e). Collectively, the Neighbor-Joining tree analysis results, expression patterns, and subcellular localization results suggest PagKNAT5a functions as a potential transcription factor involved in poplar xylem development.

### Xylem development is promoted in *PagKNAT5a* overexpression plants

**Figure 2:**
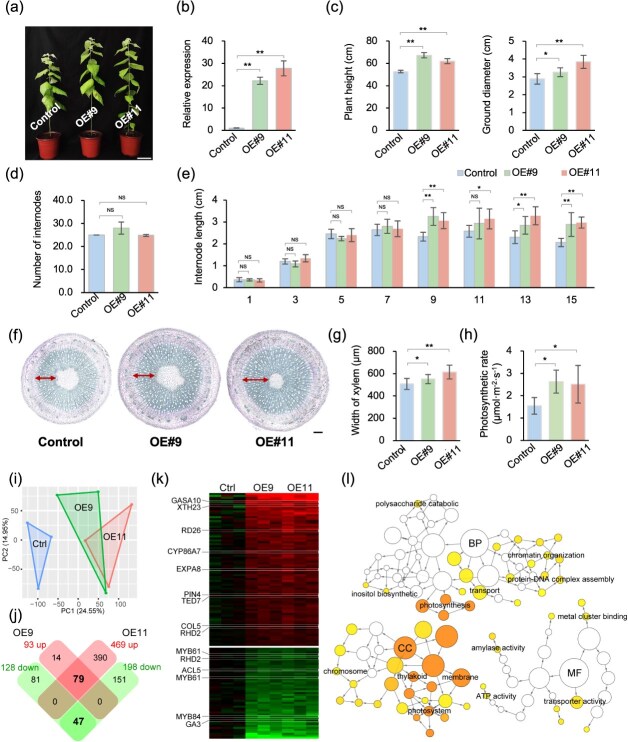
Phenotypic data of 84K plants and *pro35S:PagKNAT5a* transgenic lines. (a) Phenotypes of 2-month-old soil grown 84K plants and *pro35S:PagKNAT5a* transgenic lines. Scale bar: 10 cm. (b) Relative expression of *PagKNAT5a* in the stems of 84K plants and *pro35S:PagKNAT5a* transgenic lines. Data are represented as mean ± SD (*n* = 4). ^**^P ≤ 0.01; Student’s *t*-test. (c) Statistical data for plant height and ground diameter of 84K plants and *pro35S:PagKNAT5a* transgenic lines. Data are represented as mean ± SD (*n* = 5). ^*^P ≤ 0.05; ^**^P ≤ 0.01; Student’s *t*-test. (d) Statistical data for number of internodes from 84K plants and *PagKNAT5a* overexpression lines. Data are represented as mean ± SD (*n* ≥ 7). (e) Statistical data for the length of the 1st, 3rd, 5th, 7th, 9th, 11th, 13th, and 15th internode from 84K plants and *PagKNAT5a* overexpression lines. Data are represented as mean ± SD (*n* ≥ 7). ^*^P ≤ 0.05; ^**^P ≤ 0.01; Student’s *t*-test. (f) Cross-sections of 10th stems from 84K plants and *pro35S:PagKNAT5a* transgenic lines. Scale bar: 200 μm. (g) Statistical data for xylem width of the 10th stems from 84K plants and *pro35S:PagKNAT5a* transgenic lines. Data are represented as mean ± SD (*n* = 15). ^*^P ≤ 0.05; ^**^P ≤ 0.01; Student’s *t*-test. (h) Comparison of the photosynthetic rates between the 84K plants and *pro35S:PagKNAT5a* transgenic lines. Data are represented as mean ± SD (*n* = 3). ^*^P ≤ 0.05; Student’s *t*-test. (i) The principal components analysis (PCA) shows clear separation between the 84K plants and *pro35S:PagKNAT5a* transgenic lines. (j) DEGs overlapped in *pro35S:PagKNAT5a* transgenic lines (OE#9 and OE#11) compared to 84K plants. (k) Heatmap of core-DEGs. (l) GO enrichment analysis of core-DEGs.

To further investigate the role of *PagKNAT5a* in xylem formation, the *pro35S:PagKNAT5a* transgenic lines were constructed, and two lines that exhibited 22-fold and 28-fold increases in *PagKNAT5a* transcripts were selected for subsequent gene functional analysis (Fig. 2a and b). Phenotypic investigation of 2-month-old soil-grown plants revealed significant increases in plant height and ground diameter in *PagKNAT5a* overexpression lines compared to the control line 84K (Fig. 2c). While internode number showed no differences (Fig. 2d), the partial internode length between the 1st and 15th stems was examined, and the internode length from the 9th to 15th stems was significantly increased in the *PagKNAT5a* overexpression lines compared to the control (Fig. 2e). This suggests that increased plant height results from stem elongation. Toluidine blue O (TBO) staining of the 10th stem sections showed an 8.5% and 21.0% increase in xylem width in *PagKNAT5a* overexpressed plants relative to the control (Fig. 2f and g). Additionally, the photosynthetic rate was significantly elevated in transgenic plants compared with control line (Fig. 2h). These results indicate that *PagKNAT5a* promotes poplar xylem development. Transcriptome analysis of the 10th stem of 84K and *PagKNAT5a* overexpression lines revealed a total of 221 (93 upregulated and 128 downregulated) significant differentially expressed genes (DEGs) in OE#9 and 667 (469 upregulated and 198 downregulated) DEGs in OE#11, with 126 core-DEGs (79 upregulated and 47 downregulated) identified via examining the overlapped DEGs between two overexpression lines (Fig. 2i–l and Supplemental Table S1). Core-up-DEGs were enriched in ‘photosynthesis’ and ‘photosystem’ terms according to GO enrichment analysis (Fig. 2l). We further detected the transcription levels of some photosynthesis-related genes (*LHCB4.2*, *LHCB4.3*, *LHCB6*, and *PSBQ2*) in 1-month-old transgenic plant leaves via qRT-PCR analysis. The results demonstrated that the expression of these genes was significantly upregulated in *PagKNAT5a* overexpression plants (Fig. S3). Besides, genes involved in auxin transport or cell elongation, e.g. *PIN4*, *XYLOGLUCAN ENDOTRANSGLYCOSYLASE 23* (*XTH23*) and *GIBBERELLIC ACID STIMULATED ARABIDOPSIS 10* (*GASA10*), were upregulated in overexpression lines according to transcriptome analysis (Fig. 2k).

### Enhanced longitudinal elongation of xylem cells in *PagKNAT5a* overexpression plants

**Figure 3:**
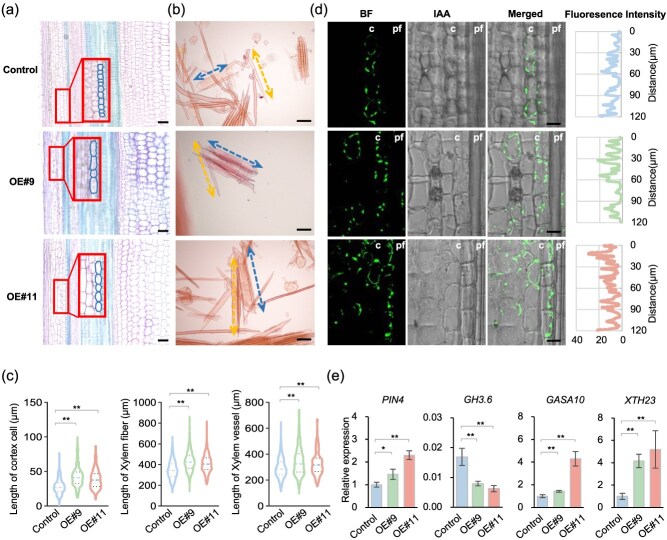
*PagKNAT5a* affects stem cell elongation. (a) Longitudinal sections of 10th stems from 84K plants and *PagKNAT5a* overexpression lines. Enlarged images shows cortex cells near the phloem fibers. Scale bar: 100 μm. (b) Safranin O staining of xylem cells from 84K plants and *PagKNAT5a* overexpression lines. Double arrows indicate vessel elements (short and wide) and fiber cells (long and narrow). Scale bar: 100 μm. (c) The lengths of cortex cell, xylem fiber, and vessel from the 10th stems of 84K plants and *PagKNAT5a* overexpression lines. At least 300 cells from five plants were used for cortex cell length measurement. Over 300 cells from 12 biological replicates were examined for vessel length analysis, and at least 1000 cells were assessed for fiber length measurements. ^*^P ≤ 0.05; ^**^P ≤ 0.01 by Student’s *t*-test. (d) Immunodetection of IAA in cortex cells near the phloem fibers of 84K plants and *PagKNAT5a* overexpression lines. c, cortex; pf, phloem fibers. Scale bar: 20 μm. The graphs on the right show the density profiles scanned over a length of 120 μm along the two layers of cortex cells attached to the phloem fibers. (e) Transcript level analysis of *PIN4*, *GH3.6*, *GASA10*, and *XTH23* in *PagKNAT5a* overexpression lines. Data are represented as mean ± SD (*n* = 3). ^*^P ≤ 0.05; ^**^P ≤ 0.01 by Student’s *t*-test.

To assess the influence of *PagKNAT5a* on stem elongation, longitudinal sections from the 10th stems of 84K plants and *PagKNAT5a* overexpression lines were analyzed. TBO staining showed a significant increase in cortex cell length near phloem fibers in *PagKNAT5a* overexpression lines (Fig. 3a and c). Disaggregation of xylem fibers and vessels revealed substantial increases in fiber cell length (23.8% and 17.7%) and vessel length (17.0% and 12.5%) in *PagKNAT5a* overexpression plants compared to controls (Fig. 3b and c), indicating that *PagKNAT5a* promotes longitudinal elongation of xylem cells.

Immunodetection of indole-3-acetic acid (IAA) showed elevated auxin signaling in cortical cells adjacent to phloem tissues (Fig. 3d) and ray cells in xylem of transgenic plants (Fig. S4). qRT-PCR confirmed increased expression of auxin efflux carrier gene *PIN4* and downregulation of IAA-amido synthase coding gene *GH3.6* in transgenic plants. *In situ* PCR hybridization revealed specific expression of these genes in phloem, xylem, and ray parenchyma cells (Fig. S5). Upregulation of *XTH23* and *GASA10*, which are responsive to auxin signaling and involved in cell elongation [26–29], was also noted (Fig. 3e), with expression signal detected in phloem and xylem of tissue-cultured 84K plants (Fig. S5). Therefore, the elevated auxin levels may induce the expression of XTH23 and GASA10, thereby promoting xylem cell elongation. These findings suggest that *PagKNAT5a* enhances the longitudinal growth of xylem cells by modulating auxin levels.

### PagKNAT5a promotes SCW thickening

**Figure 4:**
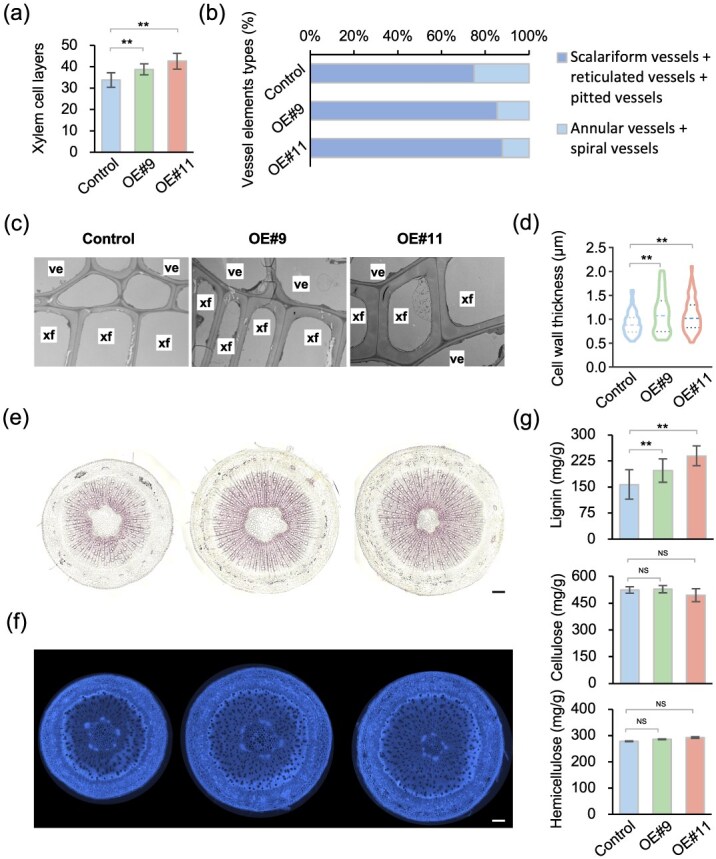
Cell wall thickness increase in *PagKNAT5a* overexpression lines. (a) Statistical data for the xylem cell layers of 84K plants and *PagKNAT5a* overexpression lines. Data are represented as mean ± SD (*n* ≥ 12). ^**^P ≤ 0.01 by Student’s *t*-test. (b) Statistical analysis of vessel elements types in xylem from 84K plants and *pro35S:PagKNAT5a* transgenic lines. Over 300 cells from 12 biological replicates were examined. (c) TEM observation of xylem fibers from 84K plants and *PagKNAT5a* overexpression lines. xf, xylem fiber; ve, vessel. Scale bar: 2 μm. (d) Statistical data for the cell wall thickness of xylem fibers from 84K plants and *PagKNAT5a* overexpression lines. Data are represented as mean ± SD. Forty cells from four biological replicates were used for cell wall thickness analysis. ^**^P ≤ 0.01 by Student’s *t*-test. (e) Phloroglucinol-HCl staining of stem sections from 84K plants and *PagKNAT5a* overexpression lines. Scale bar: 200 μm. (f) Calcofluor White staining of stem sections from 84K plants and *PagKNAT5a* overexpression lines. Scale bar: 200 μm. (g) Quantification of cellulose, lignin, and hemicellulose from stems of 84K plants and *PagKNAT5a* overexpression lines. Data are represented as mean ± SD (*n* ≥ 5). ^*^P ≤ 0.05; ^**^P ≤ 0.01 by Student’s *t*-test.

To further investigate *PagKNAT5a*-induced xylem expansion, the number of xylem cell layers and vessel types were quantified, revealing a substantial increase in both xylem cell layers and scalariform/reticulated/pitted vessels proportion in transgenic lines (Fig. 4a and b). The cell wall thickness of xylem fiber cells at different distances from cambium was measured subsequent to scanning electron microscopy (SEM). The results revealed that the SCW of fiber cells in transgenic plants was thicker than that of control plants at varying distances from the cambium (Fig. S6). Transmission electron microscope (TEM) confirmed a 16% and 20% increase in cell wall thickness for fiber cells in OE#9 and OE#11, respectively, compared to 84K poplar (Fig. 4c and d). To assess lignin and cellulose contents, phloroglucinol-HCl and Calcofluor White staining of cross-sections were performed, respectively. Phloroglucinol-HCl staining displayed a darker coloration in transgenic lines compared to that in 84K, indicating higher lignin content (Fig. 4e). However, no significant difference was observed via Calcofluor White staining (Fig. 4f). Further quantitative analysis of cell wall components revealed that lignin content in *35S:PagKNAT5a* transgenic lines was 26% and 53% greater than in 84K, with no significant differences in cellulose or hemicellulose content (Fig. 4g). These findings suggest that *PagKNAT5a* may accelerate xylem radial development, including enhancing the process of SCW thickening by promoting lignin biosynthesis.

### PagKNAT5a and MYB46 collaboratively regulate SCW thickening

**Figure 5:**
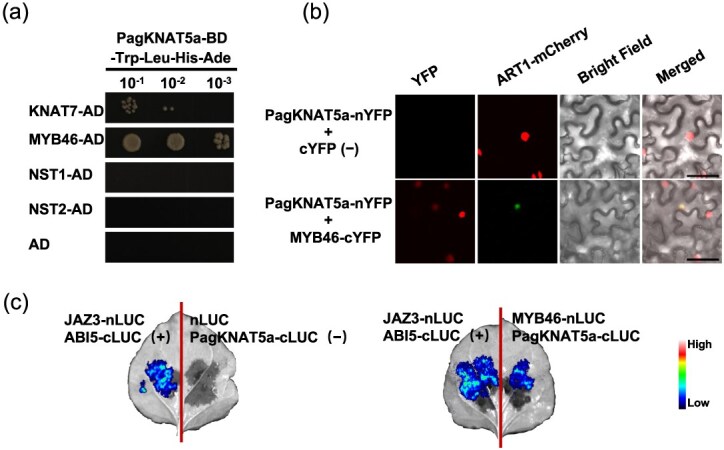
Interaction between PagKNAT5a and MYB46. (a) Interaction relations between PagKNAT5a and other master switches for SCW synthesis were examined in yeast cells. (b) Visualization of interaction relation between PagKNAT5a and MYB46 in *Nicotiana* leaves using BiFC analysis. Scale bar: 50 μm. (c) PagKNAT5a and MYB46 interact in *Nicotiana* leaves *via* split luciferase complementation experiment.

The molecular mechanism underlying *PagKNAT5a*-mediated SCW thickening was explored by screening for interacting proteins using a yeast library composed of xylem development-related transcription factors. The important SCW synthesis switches MYB46 and KNAT7 [30] were found polymerized with PagKNAT5a in yeast two-hybrid assay. However, the homologs of *Arabidopsis* NST1 and NST2, previously reported to interact with KNAT3 [19], failed to interact with PagKNAT5a (Fig. 5a), indicating a distinct SCW thickening mechanism in poplar. Given the crucial role of MYB46 in SCW synthesis, further bimolecular fluorescence complementation (BiFC) (Fig. 5b) and luciferase complement assays (Fig. 5c) verified the physical interaction between MYB46 and PagKNAT5a. The expression of MYB46 was also examined based on RNA sequencing data obtained from AspWood [23] and *in situ* PCR hybridization, revealing its widespread distribution in vascular tissue including cambium phloem and xylem, overlapping with *PagKNAT5a* expression in xylem (Figs S2 and S5). These results suggest a potential collaboration between PagKNAT5a and MYB46 in regulating SCW thickening.

### PagKNAT5a and MYB46 synergistically upregulate the expression of lignin biosynthetic genes

**Figure 6:**
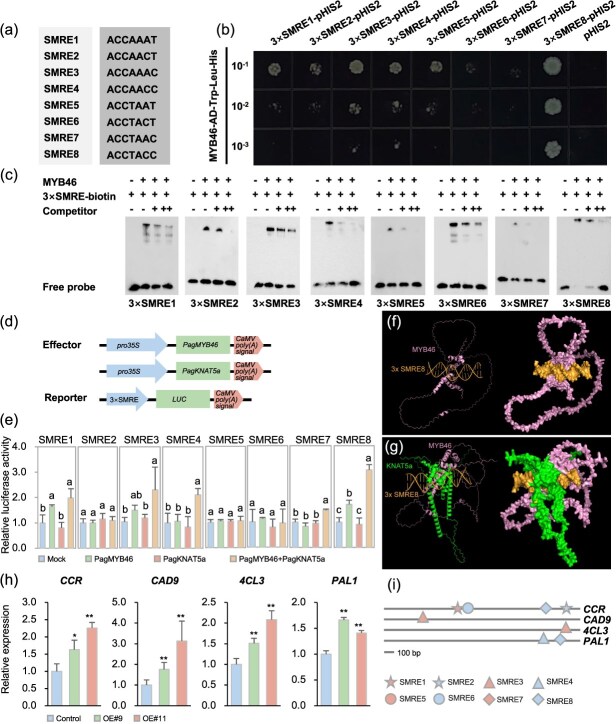
The collaboration between PagKNAT5a and MYB46 activated the expression of genes involved in lignin biosynthesis. (a) Sequences of MYB46-binding SMREs. (b) Interaction relations between MYB46 and SMREs examined in yeast cells via Y1H assays. (c) EMSA showing the binding of MYB46 to SMREs for competition analyses; unlabeled 3× SMREs fragments (competitors) were included in the reactions at 100-fold and 200-fold molar excess relative to the biotin-labeled 3× SMREs. (d) Schematic representation of the effector and reporter constructs used in transactivation assay. (e) Analysis of the activation of PagKNAT5a in the transcriptional regulation of SMREs mediated by MYB46. Transactivation activity was assessed using a relative LUC/REN ratio in the *Nicotiana* leaves. Combination of specific reporter and empty effector vector as a mock treatment. Data are represented as mean ± SD (*n* ≥ 3). The least significance difference test was applied at 0.05 probability level. (f) Simulation of the interaction between MYB46 and 3× SMRE8. (g) Simulation of the interaction effect between PagKNAT5a and MYB46 on downstream 3× SMRE8. (h) Transcript level analysis of genes involved in lignin synthesis in *PagKNAT5a* overexpression lines. Data are represented as mean ± SD (*n* = 3). ^*^P ≤ 0.05; ^**^P ≤ 0.01 by Student’s *t*-test. (i) SMREs *cis*-element analysis in the promoters of genes involved in lignin biosynthesis.

As a master switch in SCW synthesis regulation, MYB46 plays a crucial role in regulating a series of downstream transcription factors associated with SCW synthesis [30]. Eight secondary wall MYB-responsive elements (SMREs) downstream of MYB46 have been identified in *Arabidopsis* [31]. To determine whether poplar MYB46 can directly activate these SMREs (Fig. 6a), we performed yeast one-hybrid assay and found that MYB46 bound directly to all the SMREs (Fig. 6b). Incubation of different biotin-labeled 3× SMREs with the MYB46-GST fusion protein resulted in a mobility shift of the SMREs probe during electrophoretic mobility shift assay (EMSA) (Fig. 6c), confirming that MYB46 directly binds to these SMREs.

To assess the impact of PagKNAT5a-MYB46 interaction on these downstream SMREs, synthetic 3× SMREs were inserted into reporter vectors harboring the luciferase gene (LUC), while the coding sequence (CDS) of PagKNAT5a and MYB46 were cloned to construct effector vectors (Fig. 6d). Various combinations of effector and reporter constructs were co-transformed into *Nicotiana* leaves for transactivation assay. As a result, the presence of PagKNAT5a further enhanced the activation effect of MYB46 on LUC expression driven by SMRE3, SMRE4, SMRE7, and SMRE8 (Fig. 6e). Utilizing AlphaFold3 for simulating the interaction between transcription factors and *cis-*acting elements, 3D simulation results suggested that PagKNAT5a potentially enhances the binding affinity between MYB46 and the *cis*-acting element (Fig. 6f and g). This structural prediction correlates with the observed enhancement of transcriptional activation (Fig. 6e). Enzymes involved in lignin synthesis, such as 4-coumarate CoA ligase (4CL), cinnamyl alcohol dehydrogenase (CAD), phenylalanine ammonia lyase (PAL), and cinnamoyl-CoA reductase (CCR), are known to be encoded by MYB46 downstream target genes in *Arabidopsis* [32]. qRT-PCR analysis revealed transcriptional levels increase of *CCR*, *CAD9*, *4CL3*, and *PAL1* in *PagKNAT5a* overexpression lines (Fig. 6h). *Cis*-acting element analysis of the promoters of these genes revealed that each retained at least one SMRE that could be further activated by PagKNAT5a-MYB46 interaction (Fig. 6i). To complement the reporter assays, we conducted additional transcription activation assay to investigate the impact of the interaction between PagKNAT5a and MYB46 on the expression of those lignin synthesis genes (Fig. S7). As a result, PagKNAT5a enhances the transcription activation of MYB46 on *CCR*, *CAD9*, *4CL3*, and *PAL1*. The results suggests that the upregulation of these lignin synthesis genes is synergistically mediated by PagKNAT5a and MYB46 through the activation of downstream SMREs.

## Discussion

It is intriguing that despite their close phylogenetic relationship (Fig. S1a), KNAT7 exhibited distinct biological functions compared to PagKNAT5 and KNAT3. Motif and domain analysis of class II KNOX family members (Fig. S1b) reveals that KNAT7 lacks the conserved ELK domain present in other class II KNOX family members. Consequently, PagKNAT5a shares greater similarity with KNAT3 and KNAT4 based on domain characterization. The ELK domain, proposed to form an amphipathic helix [33], is implicated in nuclear localization or binding to specific DNA sequences [34, 35] and is considered a protein–protein interaction domain [36]. Variations in the ELK domain may lead to differences in interacting proteins, resulting in functional diversity. Existing evidence suggests that KNAT7 is more likely to negatively regulate SCW synthesis by interacting with BLH6, MYB75, and NAC31 [13, 18, 20–22], while KNAT3 tends to promote SCW synthesis by interacting with SCW-positive regulators NST1 and NST2 to promote the expression of *F5H*, which regulates lignin synthesis [19]. This structural similarity between PagKNAT5a and KNAT3 suggests that PagKNAT5a may also promote SCW synthesis. However, PagKNAT5a does not interact with either NST1 or NST2 (Fig. 5a). Thus, although PagKNAT5a promoted SCW thickening in poplar (Fig. 4c and d**;**  Fig. S6), it may have different collaborators.

Early studies indicated that KNAT7 belongs to the third layer of the SCW synthesis regulatory network, functioning downstream of MYB46 [30]. However, subsequent research has demonstrated that class II KNOX gene family members also interact with top-level master switches such as NAC31, NST1, and NST2 [13, 19], suggesting that class II KNOX gene family members function flexibly in SCW synthesis. MYB46, a master switch for SCW synthesis in *Arabidopsis*, is specifically expressed in fiber cells and vessels [37]. Dominant repression of *MYB46* resulted in reduced SCW thickness in these cell types, while its overexpression promotes SCW deposition by activating the expression of genes involved in cellulose, xylan, and lignin biosynthesis [37, 38]. Lignin provides mechanical strength to plant cell walls, which is the most important component and utilization part of wood [39, 40]. In this study, the interaction between PagKNAT5a and MYB46 has been confirmed via yeast two-hybrid assay, BiFC, and luciferase complementation test (Fig. 5). Furthermore, we observed elevated transcription levels of lignin synthesis-related genes, including *CCR*, *CAD9*, *4CL3*, and *PAL1*, in *PagKNAT5a* overexpression lines (Fig. 6h). In both *Arabidopsis* and poplar, MYB46 has been identified to directly bind to the SMRE sequences in the promoter regions of its downstream genes, with the core structure being (T/C)ACC(A/T)A(A/C)(T/C) [38, 41]. The promoter regions of these genes contain SMRE3, SMRE4, or SMRE8 (Fig. 6i), which can be activated through the interaction between PagKNAT5a and MYB46 (Fig. 6d–g). These results suggest that PagKNAT5a promotes lignin accumulation (Fig. 4e and g) by upregulating the expression levels of downstream MYB46 target genes *CCR*, *CAD9*, *4CL3*, and *PAL1*, and thus facilitates SCW deposition in xylem fiber cells (Fig. 4c and d). The formation process of secondary xylem cells includes the initial proliferation and differentiation of cambium cells, followed by the deposition of the SCW and programmed cell death [42]. *PagKNAT5a* overexpression plants exhibited significantly thicker fiber cell SCWs at different distances from the cambium compared with control plants (Fig. S6), indicating that PagKNAT5a may promote xylem development (Fig. 2f and g) by facilitating the formation of the fiber cell SCW, which constitutes the structure of woody plant biomass [43].

Class I KNOX transcription factors are known to be involved in multiple hormone pathways including auxin signaling [44]. Scanlon *et al.* [45] demonstrated a connection between polar auxin transport and the expression of KNOX genes. KN1 directly regulates genes involved in the synthesis, transport, and signaling of auxin in *Zea mays* [46]. Overexpression of *Tkn4* in *Solanum lycopersicum* plants resulted in increased sensitivity to auxin, accompanied by upregulation of genes involved in IAA synthesis and transport, including *SlPIN3*, *SlPIN9*, *GH3.8*, and *ARF9* [47]. Recent studies have also revealed that class I KNOX members promote axillary bud development in chrysanthemums and poplar by blocking auxin synthesis pathways [48, 49]. However, research on the role of class II KNOX members in auxin regulation remains limited. In our study, we found that overexpression of *PagKNAT5a* resulted in an upregulation of the auxin efflux carrier encoding gene *PIN4* and a significant decrease in *GH3.6* expression (Fig. 3e). The *Arabidopsis GH3.6* homologous gene, *DFL1*, encodes an IAA-amido synthase that negatively regulates shoot cell elongation by conjugating Asp to IAA, leading to the degradation of IAA-Asp. Antisense transgenic lines of *DFL1* exhibited larger shoots [50, 51]. The results suggest that PagKNAT5a enhances auxin levels in vascular tissues (Fig. 3d; Fig. S4) by regulating genes involved in auxin synthesis and transport, which may activate the downstream response factors, i.e. *GASA10* and *XTH23* (Fig. 3e), which have been demonstrated to respond to IAA and promote cell elongation in *Arabidopsis* and cotton, respectively [26, 52]. Consequently, longitudinal expansion was observed in vascular cells within *PagKNAT5a* overexpression transformants (Fig. 3a–c), leading to an increase in plant height (Fig. 2a, c, and e). These results indicate a potential role for class II KNOX members in hormone regulation.

Research on the correlation between KNOX family members and plant photosynthesis has been limited. However, our findings indicate that overexpression of *PagKNAT5a* significantly enhances the photosynthetic rate (Fig. 2h). The qRT-PCR analysis revealed the involvement of PagKNAT5a in the regulation of genes related to photosynthesis (Fig. S3). This enhanced photosynthesis in overexpressing plants may provide substantial support for both vertical and horizontal xylem development.

## Conclusion

**Figure 7:**
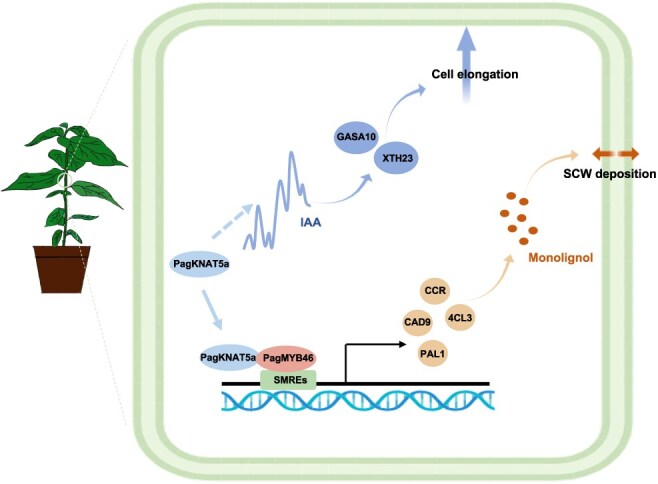
Schematic model of PagKNAT5a-mediated xylem cell elongation and secondary cell wall formation. PagKNAT5a enhances longitudinal cell elongation in vascular tissues by modulating auxin accumulation. Furthermore, its physical interaction with MYB46 synergistically upregulates key genes involved in the lignin biosynthesis pathway.

In conclusion, PagKNAT5a plays a crucial role in regulating plant development in poplar by facilitating the longitudinal elongation of vascular cells through modulation of auxin. Additionally, it enhances the thickness of fiber cell SCW by activating MYB46-mediated downstream genes involved in lignin biosynthesis, ultimately contributing to improved biomass in poplar trees (Fig. 7). The functional investigation of this gene reveals the potential to simultaneously enhance photosynthesis and promote both longitudinal and radial growth in poplar trees, thereby offering promising applications for increasing wood yield in forestry varieties.

## Materials and methods

### Transgenic lines construction and culture conditions

The entire coding sequence of *PagKNAT5a* was cloned from 84K and inserted into binary vectors pMDC32 and pMDC43 to generate the *pro35S:PagKNAT5a* and *pro35S:PagKNAT5a-GFP* plasmids, respectively. The 2 kb native promoter of *PagKNAT5a* was amplified from 84K and introduced into binary vector pMDC164 to create the *proPagKNAT5a:GUS* construct. These constructs were transformed into 84K calli through *Agrobacterium*-mediated transformation [8]. Primer sequences are listed in Supplemental Table S2.

The 84K plants and *35S:PagKNAT5a* transgenic lines were maintained and propagated through tissue culture at 25°C under a photoperiod of 16-h light and 8-h dark. One-month-old young plants were transferred from the culture media to soil and grown for an additional 2 months under the same temperature and photoperiod conditions prior to phenotypic investigation and various physiological tests.

### Transcript level analysis

Total RNA was isolated using an RNA extraction kit (NG3021S, HLINGENE) and reverse transcribed with a reverse transcription kit (AG11705, Accurate). qRT-PCR analysis was performed using 2× SYBR Green Pro Taq HS Premix IV (AG11746, Accurate) on a QuantStudio 3 Real-Time PCR System (Thermo Fisher Scientific, Wilmington, DE, USA). For analysis of *PagKNAT5a* expression patterns, the first to third unfolding leaves, roots, and root tips and the first to ninth internodes were collected from 1-month-old soil-grown 84K plants. To assess transcriptional levels of *PagKNAT5a* and lignin synthesis-related genes in transgenic plants, the first to ninth internodes were harvested from 1-month-old soil growing 84K plants and transgenic lines, respectively. Tests were repeated at least three times.

### Histochemical staining

For vascular morphology observation, the 10th internodes from 2-month-old soil grown plants were harvested and sectioned at a thickness of 50 μm using a vibratome (VT1200S; Leica, Wetzlar, Germany). Sections were then infiltrated in 0.1% (w/v) TBO prior to microscopy (DM6B, Leica) [53]. At least 15 sections collected from five plants were used for measuring xylem width and cortex cell length, and the analysis was repeated three times. To roughly determine lignin and cellulose contents, sections were stained with 1% phloroglucinol (w/v)/HCl for 5 min and 0.01% (v/v) Calcofluor White (18 909; Sigma) for 3 min, respectively, before observation. The microscopy image acquisition parameters remained consistent across all slides during the same test. To investigate xylem element types and lengths, the 10th stems were harvested, bark peeled, infiltrated in a solution prepared by the reaction of (30%) hydrogen peroxide and glacial acetic acid with a ratio of 1:1 (v/v), followed by heating in a water bath at 65°C for sufficient dissociation, and centrifuged at 5000 rpm for 10 min; the precipitate was washed with ddH_2_O and stained with Safranin O before microscopy.

To perform GUS activity analysis, 1-month-old tissue-cultured stable *proPagKNAT5a:GUS* transgenic plants were harvested and fixed in 90% acetone for 1 h; samples were washed three times with washing buffer (0.2% Triton X-100, 2 mM K_3_[Fe(CN)_6_], 2 mM K_4_[Fe(CN)_6_], 50 mM phosphate buffer, pH 7.0), followed by infiltration in staining solution (1 mg/mL X-Gluc in washing buffer) under vacuum for 1 h. The samples were then incubated overnight at a temperature of 37°C before being immersed in 70% ethanol for decoloration and then imaged. Samples were sliced to a thickness of 50 μm for microscopic examination (DM6B, Leica) to assess GUS expression in vascular tissues.

### Cell wall composition analysis

The 7th–15th stems from 2-month-old soil-grown 84K plants and *PagKNAT5a* overexpression lines were collected and bark peeled for cell wall composition determination. Relative quantification of lignin, cellulose, and hemicellulose was performed using the acetyl bromide method (M1711A, Suzhou Michy Biomedical Technology), the anthrone assay (M1718A, Suzhou Michy Biomedical Technology), and the 3,5-dinitro salicylic acid colorimetric method (M1719A, Suzhou Michy Biomedical Technology), respectively. The absorbances of the samples were measured by enzyme-linked immunosorbent assay (ELISA) (SPARK; TECAN, Switzerland) at wavelengths of 540, 340, and 620 nm, respectively.

### Immunohistochemistry

For IAA immunodetection, the 10th stems from 2-month-old plants were harvested and fixed in 4% formaldehyde (freshly depolymerized from paraformaldehyde) for 30 min at room temperature. Longitudinal slices at a thickness of 50 μm were prepared and immersed in 1× phosphate buffered saline (PBS) containing 0.1% Tween for a duration of 30 min. The sections were then blocked with blocking buffer containing 5% milk in 1× PBS for 1 h. Then the primary antibody rabbit anti-IAA-N1 (AS09421, Agrisera) was applied at a dilution of 1:100 and incubated overnight at 4°C. After three washes with 1× PBS (each time for 10 min), the sections were treated with secondary DyLight®488-conjugated goat anti-rabbit IgG antibody (AS09633, Agrisera) diluted to a ratio of 1:100 in blocking buffer for 1 h and observed using a confocal laser scanning microscope (FV3000; Olympus, Tokyo, Japan) at 488 nm for excitation and 550 nm for emission with the same image capture settings. Immunodetection of IAA in the cross-sections of the 10th stem was performed to analyze auxin level in xylem and observed using an upright microscope (DM6B, Leica) at 460 to 500 nm for excitation and 512 to 542 nm for emission [53].

### Transmission electron microscopy

Transmission electron microscopy was conducted by first trimming the 10th stems from both transgenic lines and 84K control into small segments. These were then submerged in a precooled fixing solution (2.5% glutaraldehyde in 0.1 M phosphate buffer, pH 7.0), followed by washing thrice with 0.1 M phosphate buffer (pH 7.0). Subsequently, they were placed in a 2% (w/v) osmic acid solution for an overnight soak. The dehydration process involved a sequential ethanol series (50%, 70%, 80%, 95%), followed by acetone treatment. The samples were then embedded in resin, sliced into sections, dyed, and finally examined using a TEM (model H-7650; Hitachi, Tokyo, Japan).

### Yeast two-hybrid assays

The CDS of *PagKNAT5a* was cloned from hybrid poplar 84K and introduced into vector pGBK7 to generate bait constructs. A prey library containing 227 plasmids encoding xylem development-related transcription factors was utilized for screening interacting proteins with PagKNAT5a. Bait and prey plasmids were co-transformed into AH109 yeast strain, which were subsequently screened in SD-Trp-Leu-His-Ade medium (PM2112, Coolaber).

### BiFC assay and luciferase complementation test

BiFC was performed as previously described [54]. The CDS of *PagKNAT5a* and *MYB46* were amplified from hybrid poplar 84K and introduced into SPYNE(R) and SPYCE(M) vectors, respectively. The resulting constructs were then transformed into *Agrobacterium* strain GV3101 for co-infiltration into *Nicotiana* leaves. After incubation in darkness for 1 day followed by 2 days of light exposure, yellow fluorescent protein signals were detected using a confocal laser scanning microscope (FV3000, Olympus) with excitation at 488 nm and emission at 520 nm. mCherry signals were captured with excitation at 561 nm and emission at 595 nm. For luciferase complementation assays [19], the CDS of *PagKNAT5a* and *MYB46* were cloned and introduced into vectors pCAMBIA1300-cLUC and pCAMBIA1300-nLUC vectors, respectively. Following infection of *Nicotiana* leaves as described above, the leaves were sprayed with 150 μg/ml d-luciferin (D12505, Lablead) and kept in darkness for 10 min before imaging using a chemiluminescent imaging system (Tanon-5200; Tanon Science and Technology, Shanghai, China).

### Yeast one-hybrid assays

The CDS of *MYB46* was cloned from 84K and fused into the pGADT7-Rec2 vector to generate the bait construct. 3× SMREs were synthesized and introduced into the pHIS2 vector to generate the prey construct. Bait and prey plasmids were co-transformed into the Y187 yeast strain, followed by screening on SD-Trp-Leu-His medium (PM2152, Coolaber) supplemented with 10 mM 3-amino-1,2,4-triazole (3-AT).

### Electrophoretic mobility shift assay

The *MYB46* CDS was amplified and cloned into the pGEX-4T-1 vector to generate an expression vector for the MYB46-GST fusion protein. After transforming the construct into *Escherichia coli* strain BL21(DE3) (EC1002, WEIDI), expression of the target protein was induced with 0.5 mM IPTG at 16°C with shaking at 200 rpm overnight. The purified fusion protein was incubated with biotin-labeled 3× SMREs using a protein-DNA binding interaction assay kit (20 148, Thermo Fisher Scientific) at 25°C for 20 min. Following separation by polyacrylamide gel electrophoresis, the reactant products were electroblotted onto a nylon membrane and detected using a chemiluminescent nucleic acid detection module kit (89 880, Thermo Fisher Scientific).

### Transactivation assay

The dual-luciferase reporter transient transactivation assay was conducted according to a previously published protocol [19]. Effector plasmids were generated by cloning and inserting the CDS of *PagKNA5a* and *MYB46* into the pGreen II 62-SK vector, respectively. Reporter plasmids were constructed by fusing synthesized 3× SMREs into pGreen II 0800-LUC vectors. Various combinations of effector and reporter constructs were co-transformed into *Nicotiana* leaves following the same procedure as described above. The infected leaves were collected, homogenized in 1× passive lysis buffer (PLB) buffer, and centrifuged. The supernatant was subsequently subjected to LUC and REN activity assays using a Dual-Luciferase Reporter Assay System (E1910; Promega, Wisconsin, USA) and detected (GloMax2020, Promega). The LUC/REN ratio indicates transcriptional activity.

### *In situ* PCR hybridization

The seventh internode of 1-month-old tissue-cultured 84K plant was fixed (63% ethanol, 5% acetic acid, 2% formaldehyde) and subjected to vacuum infiltration. The samples were then embedded in 5% agarose and sectioned at a thickness of 40 μm. Recombinant DNase I (2270A, Takara) treatment was performed, followed by cDNA synthesis using SMART MMLV Reverse Transcriptase (639 523, Takara). *In situ* PCR was carried out using PrimeSTAR® Max DNA Polymerase (R047A, Takara) and digoxigenin (DIG)-labeled deoxyribonucleotide (11 093 088 910, Roche) on a Thermal Cycler (T100; BIO-RAD, California, USA). Specific primers used are listed in Supplemantal Table S2. The sections were then washed twice with 1× PBS (pH 7.5) and blocked in 1× Blocking Solution (1% bovine serum albumin in 1× PBS) for 30 min. Anti-DIG antibody conjugated to alkaline phosphatase (AP) (11 093 274 910, Roche) was added at a dilution of 1:500 and further incubated for 1 h, followed by two washes with wash buffer (0.1 M Tris-Cl, 0.15 M NaCl, pH 9.5). AP substrate (11442074001, Roche) was applied for staining for 10 min before microscopy (DM6B, Leica) [55].

### Bioinformatics analysis

The expression pattern of the *KNAT5a* gene in different tissues and different cell types was analyzed based on data obtained from the NCBI gene expression database (GSE81077) and Aspwood (http://aspwood.popgenie.org/aspwood-v3.0/). The 10th internode of 84K and *PagKNAT5a* overexpression plants were harvested for RNA extraction, RNA-Seq libraries generation, and sequencing according to a previously published protocol [56]. RNA-Seq analysis was performed using a cutoff of FoldChange >1 and false discovery rate (FDR) corrected *P* < 0.05 for the screening of DEGs [56, 59]. GO enrichment analysis was performed using agriGO [57]. The RNA-Seq data have been uploaded to the National Genomics Data Center (https://bigd.big.ac.cn/bioproject) with the BioProject accession number CRA020529. For structure predictions, we submitted the DNA sequences of SMREs and the protein sequences of PagKNAT5a and MYB46 to the AlphaFold3 server (https://alphafoldserver.com). Predictions were carried out using the server’s default parameters to ensure consistency and reproducibility in the structural analysis. The predicted model with the highest confidence (ranked 0 on a scale of 0–4) was selected as our representative model for further comparative analysis and plotting.

## Supplementary Material

Web_Material_uhaf125

## Data Availability

The RNA-Seq data has been uploaded to National Genomics Data Center (https://bigd.big.ac.cn/bioproject) with the BioProject accession number: CRA020529.
