# Supplementary material for: PagKNAT5a promotes plant growth by enhancing xylem cell elongation and secondary wall formation in poplar
Source: Hortic Res. 2025 May 7;12(8):uhaf125. doi: 10.1093/hr/uhaf125 (PMC12261911; doi:10.1093/hr/uhaf125)
Supplement: Web_Material_uhaf125 [file web_material_uhaf125.zip › Supplemental Figures.pdf]

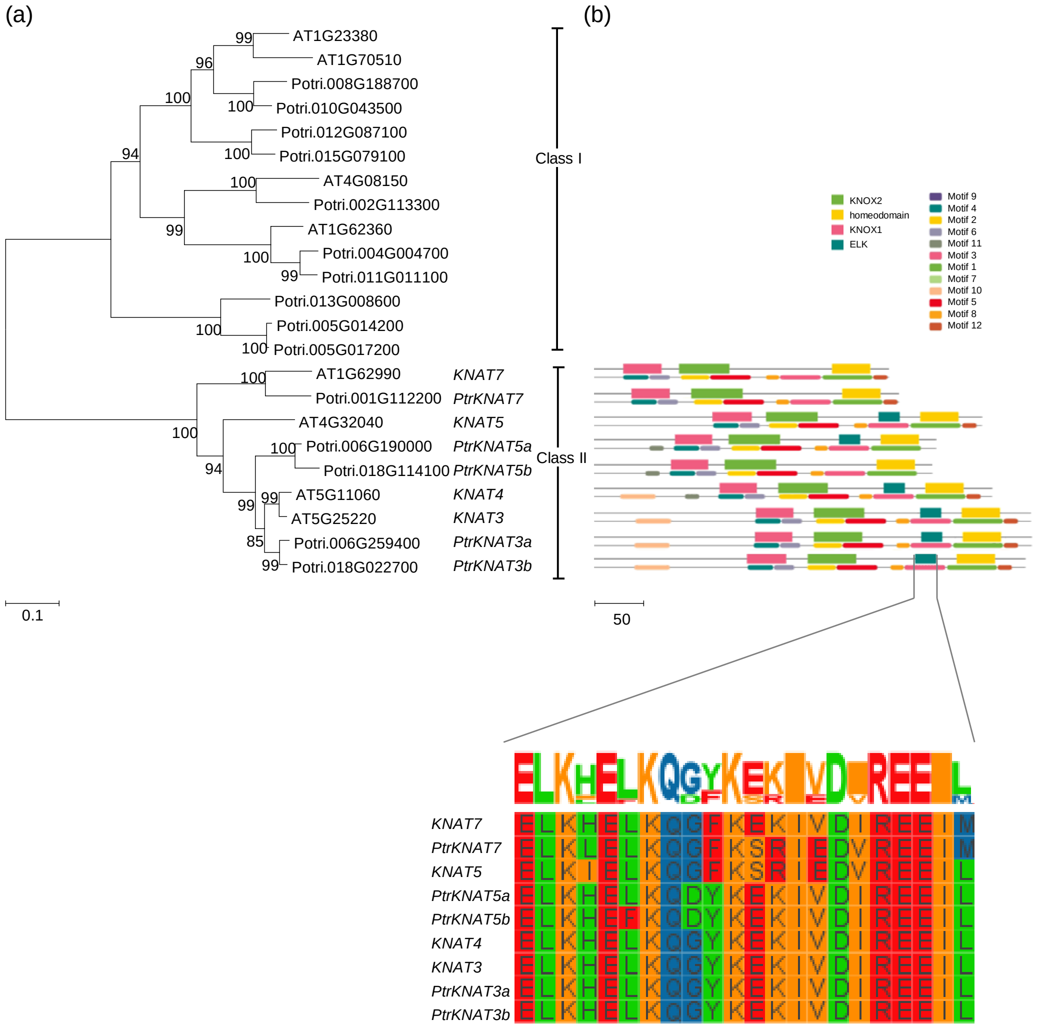

Supplemental Figure S1. Evolutionary relationship of KNOX transcription family members. (a) Neighbor-Joining tree depicting the evolutionary relationships of *Arabidopsis* and *Populus* KNOX transcription family members, constructed based on amino acid sequences alignments using MEGA 5 software. (b) Characterization of the Domain and motif structure of class II KNOX transcription factor family members.

Negative *PagKNAT5a*_P1 *PagKNAT5a*_P2

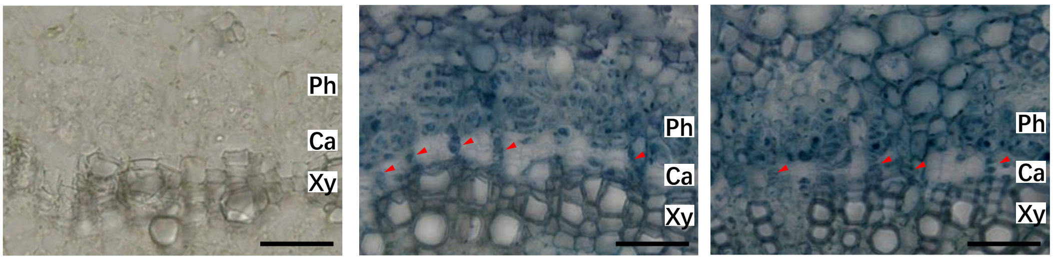

Supplemental Figure S2. *PagKNAT5a* expression in the vascular tissues. *In situ* PCR detection of *PagKNAT5a* transcripts in the 7th internode of one-month-old tissue cultured 84K plants. Ph, Phloem. Ca, Cambium. Xy, Xylem. Scale bar: 50 μm.

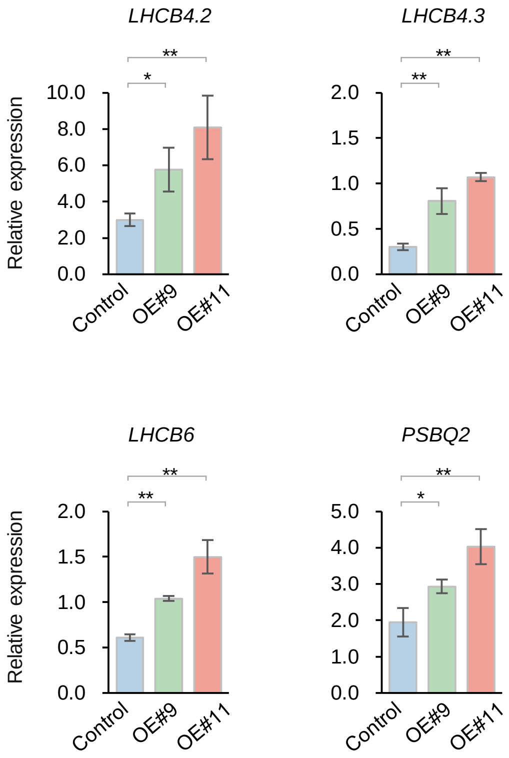

Supplemental Figure S3. Transcription levels of genes related to photosynthesis.

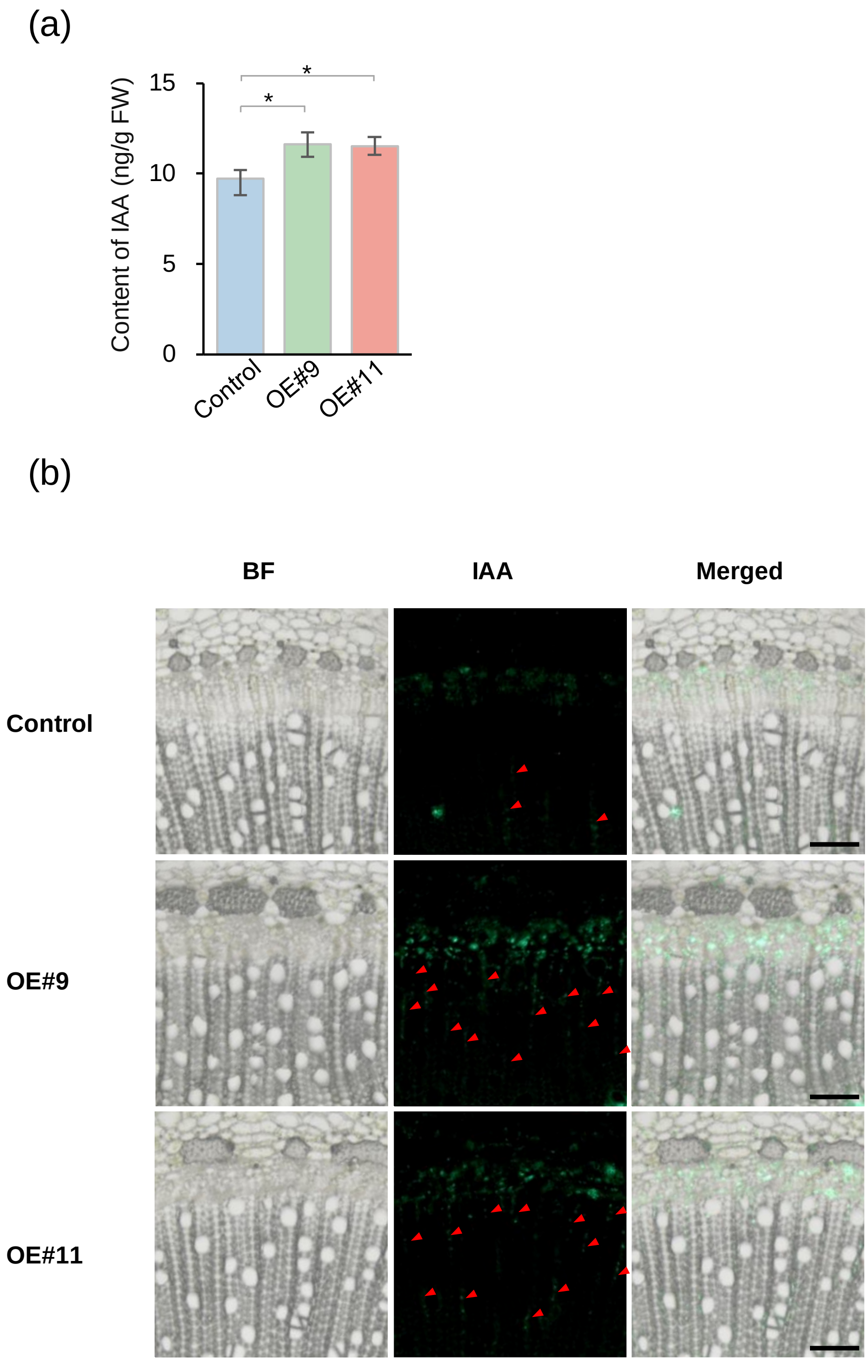

Supplemental Figure S4. *PagKNAT5a* enhances auxin levels in xylem. (a) Auxin quantitative detection of auxin content in the 10th-12th internodes from two-month-old soil grown *PagKNAT5a* overexpression plants. Data are represented as mean ± SD (n=3). *$P \leq 0.05$ by Student's t-test. (b) Immunodetection of IAA in the 10th stem of 84K plants and *PagKNAT5a* overexpression lines. The red triangle point to IAA signal detected in the ray cells. Scale bar: 100 μm.

(a)

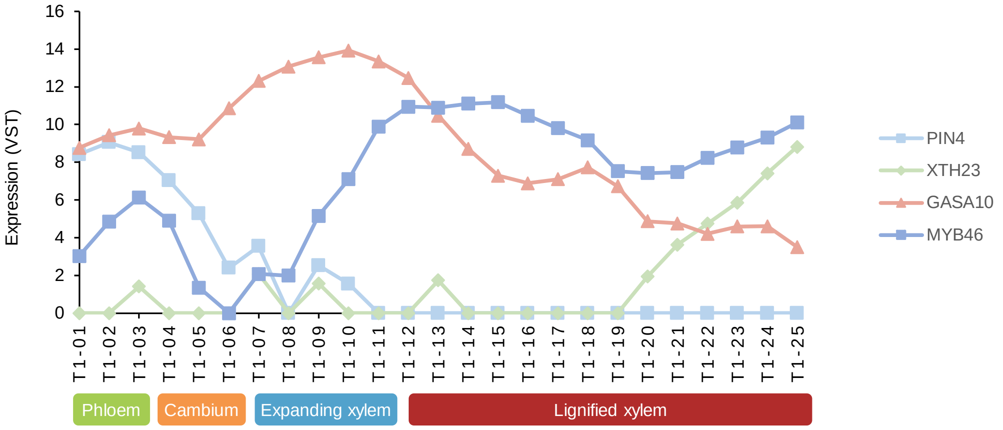

(b)

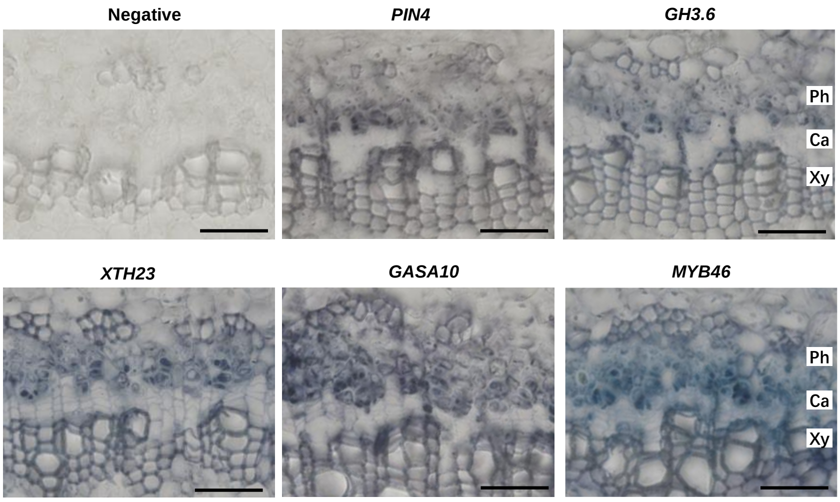

Supplemental Figure S5. Expression patterns of *PIN4*, *GH3.6*, *GASA10*, *XTH23* and *MYB46*. (a) Expression patterns of *PIN4*, *GASA10*, *XTH23* and *MYB46* in *Populus tremula* (Sundell *et al*., 2017). (b) *In situ* PCR detection of *PIN4*, *GH3.6*, *GASA10*, *XTH23* and *MYB46* transcripts in the 7th internode of one-month-old tissue cultured 84K plant. Scale bar: 50 μm.

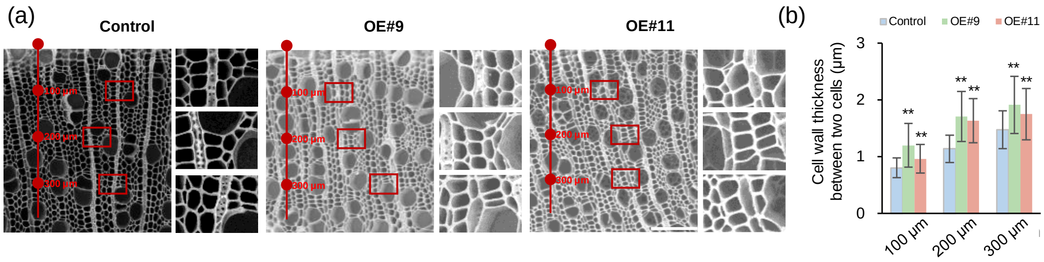

Supplemental Figure S6. Cell wall thickness analysis of *pro35S:PagKNAT5a* transgenic lines. (a) Scanning electron microscopy of stem sections from 84K and *pro35S:PagKNAT5a* transgenic lines. Images on the right show magnified views of xylem at distances of 100 μm, 200 μm and 300 μm from cambium zone respectively. Scale bar: 100 μm. (b) Statistical analysis of the fiber cell wall thickness from 84K plants and *pro35S:PagKNAT5a* transgenic lines. Data are represented as mean ± SD. At least 58 cells from four biological replicates were used for cell wall thickness analysis. $^{**}P \leq 0.01$ by Student's *t*-test.

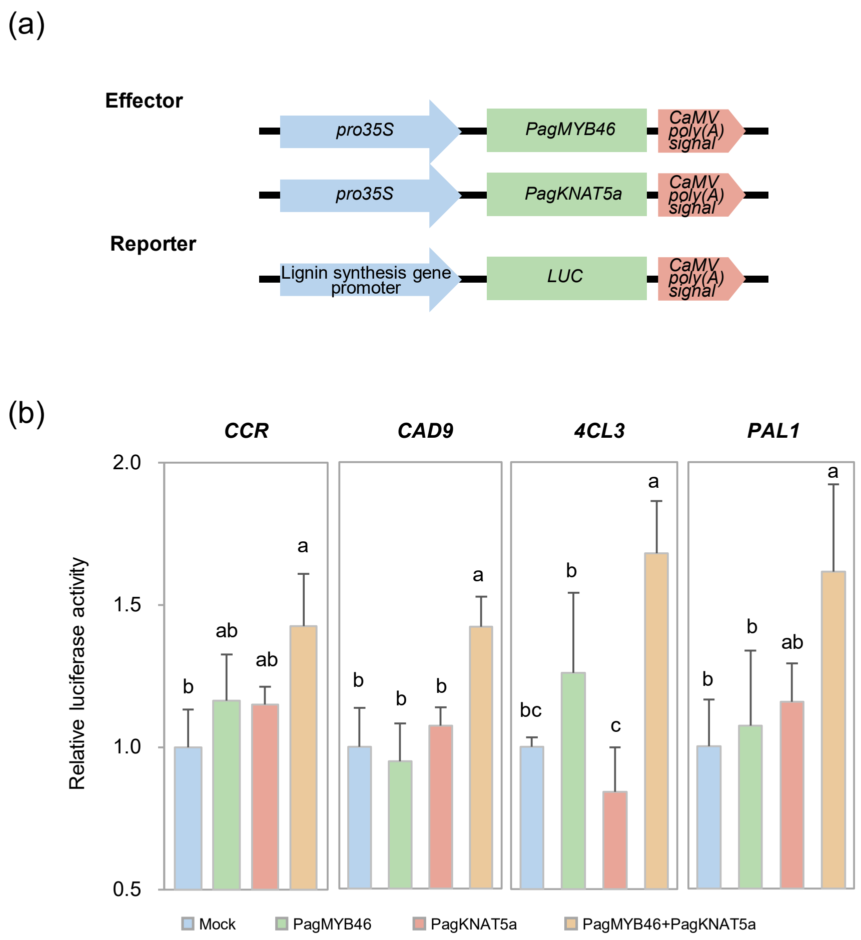

Supplemental Figure S7. Activation of lignin biosynthesis genes through interaction between MYB46 and PagKNAT5a. (a) Schematic representation of the effector and reporter constructs used in the transactivation assay. (b) Activation of lignin biosynthesis genes was evaluated by measuring the LUC/REN ratio in the *Nicotiana* leaves. Data are represented as mean ± SD ($n \geq 4$). The least significance difference test was applied at 0.05 probability level.
